# Lifespan Analyses of Forest Raptor Nests: Patterns of Creation, Persistence and Reuse

**DOI:** 10.1371/journal.pone.0093628

**Published:** 2014-04-09

**Authors:** María V. Jiménez-Franco, José E. Martínez, José F. Calvo

**Affiliations:** 1 Departamento de Ecología e Hidrología, Universidad de Murcia, Murcia, Spain; 2 Bonelli’s Eagle Study and Conservation Group, Murcia, Spain; University of Lleida, Spain

## Abstract

Structural elements for breeding such as nests are key resources for the conservation of bird populations. This is especially true when structural elements require a specific and restricted habitat, or if the construction of nests is costly in time and energy. The availability of nesting-platforms is influenced by nest creation and persistence. In a Mediterranean forest in southeastern Spain, nesting-platforms are the only structural element for three forest-dwelling raptor species: booted eagle *Aquila pennata*, common buzzard *Buteo buteo* and northern goshawk *Accipiter gentilis*. From 1998 to 2013, we tracked the fate of 157 nesting-platforms built and reused by these species with the aim of determining the rates of creation and destruction of nesting-platforms, estimating nest persistence by applying two survival analyses, describing the pattern of nest reuse and testing the effects of nest use on breeding success. Nest creation and destruction rates were low (0.14 and 0.05, respectively). Using Kaplan Meier survival estimates and Cox proportional-hazards regression models we found that median nest longevity was 12 years and that this was not significantly affected by nest characteristics, nest-tree dimensions, nest-builder species, or frequency of use of the platform. We also estimated a transition matrix, considering the different stages of nest occupation (vacant or occupied by one of the focal species), to obtain the fundamental matrix and the average life expectancies of nests, which varied from 17.9 to 19.7 years. Eighty six percent of nests were used in at least one breeding attempt, 67.5% were reused and 17.8% were successively occupied by at least two of the study species. The frequency of nest use had no significant effects on the breeding success of any species. We conclude that nesting-platforms constitute an important resource for forest raptors and that their longevity is sufficiently high to allow their reuse in multiple breeding attempts.

## Introduction

Bird nests, such as tree cavities or nesting-platforms, are key breeding structures for the dynamics and conservation of some bird populations [1], [2]. Most species depend on these resources for nesting and roosting, but some species lack the ability to create them (e.g. 55 non-excavator birds rely on tree cavities in Argentina [3]). However, when nests are available during different breeding periods throughout the years, they may be reused or expropriated by others [4]. Nests also help researchers to monitor populations [1], [5] and have an important role in management and conservation actions [6]. Moreover, when there is a shortage of nests for populations [7], effective management and conservation strategies may be necessary to ensure population viability [8], [9]. An analysis of the lifespan of nests in the context of population conservation will help determine whether a given pool of nests is sufficient for species that depend on these resources.

Longevity or survival studies have mainly been developed to assess population growth rates and the age-specific properties of individual populations of many species [10]–[13]. However, longevity studies can also be performed considering breeding structures, although such studies are less common. Recently, longevity studies have been developed using tree cavity nests, which are key elements for a wide range of species (from insects to different vertebrates) [2], [3], [14]. The longevity of these tree cavities may vary from 5 to 16 years depending on factors such as forest type, the kind of tree species, the characteristics of nest trees, the bird species using them (excavator *vs*. non-excavator species) [2], [14], as well as disturbance events affecting the study area, e.g. timber harvesting [9]. Studies focusing on the longevity of other kinds of structures, such as nesting-platforms and open-cup nests, are less common although their lifespans are known to vary widely. Whereas the nest-sites of gyrfalcons *Falco rusticolus* in cliffs may remain unaltered for hundreds of years [15], European magpie *Pica pica* nests used by forest raptors have an average survival time of around 3 years [1], while the open cup-nests of passerines deteriorate between seasons, preventing most nests from being reused [16], [17]. Despite the different longevities of these nests, they are important reproductive resources for birds, probably acting as location cues for nest-site selection [16]. Such nest-sites have been even called “ecological magnets” when they are reused over the long term [15], [18]. Nesting-platforms created and occupied by forest-dwelling raptors may also be key structures in the breeding establishment of raptor populations [19], [20]. These nests are usually large platforms constructed of twigs and leaves, and placed either between the trunk and the branches or on the branches of trees [21], [22]. Nesting-platform size is related to bird species size [23]. However, nest sizes are quite variable because birds place fresh building material to repair them during the courtship period [20], [23]. These structures may last for a number of years and be reused by several species other than the builders [20]. Therefore, a knowledge of longevity of large nesting-platforms is useful since: 1) nests are one of the most important elements for the reproduction of forest raptors because they can be used for several generations [24], 2) nests can be occupied alternately by different species in different years [25], 3) there is competition for establishment in breeding sites [26], [27], 4) the potential sites for nests is reduced since they can only be constructed in large trees [28] and therefore 5) the availability of suitable nesting structures may be limited by timber harvesting in managed forest landscapes [6], [21].

This is the first study to estimate the lifespan of nests created by a forest raptor community with inter-specific competition for nesting-platform sites in different breeding periods [29]. The community in question consists of the booted eagle *Aquila pennata* (Gmelin, 1788), the common buzzard *Buteo buteo* (Linnaeus, 1758) and the northern goshawk *Accipiter gentilis* (Linnaeus, 1758). The nesting-platforms studied are situated in a Mediterranean forest ecosystem in southeastern Spain, the Special Protection Area “Sierras de Burete, Lavia y Cambrón”, where the only tree species available for supporting nests is Aleppo pine (*Pinus halepensis*). The aims of this study were: 1) to determine the rates of construction and loss of nesting-platforms, 2) to estimate nest persistence by using two types of lifespan analysis, 3) to describe the pattern of nest reuse and alternation by different forest raptor species and 4) to assess the effects of nest use on breeding success. Kaplan-Meier estimates and Cox proportional-hazards regression models allowed us to quantify the median survival rates for nests and to test whether platform persistence varied with nesting-platform height, nest-tree height, nest-tree diameter, crown cover of the nest-tree, the nest builder species and the frequency of platform use. We expected to find that nesting-platforms would be more persistent when located in a less accessible place of the tree (i.e. a greater nest height) and in a more resistant tree (i.e. a higher nest-tree height, nest-tree diameter and nest-tree crown cover). We also thought that the longevity of nesting-platforms would depend on the nest builder species (booted eagle, common buzzard or northern goshawk). Moreover, it was thought that nesting-platforms with a higher frequency of use would be more resistant since they will have been repaired during more breeding attempts [5], giving them greater structural consistency and resistance to perturbations. We developed a transition matrix among the occupancy stages of the nesting-platforms throughout the study period to determine the life expectancy of the nesting-platforms for each occupancy stage [11].

## Materials and Methods

### Ethics Statement

Authorization for the study was provided by the Dirección General de Medio Ambiente of the Autonomous Community of Murcia, which has the duty to regulate the conservation and management of wildlife and endangered species. The entire study, which forms part of a study of forest raptor populations in the study area [22], [29]–[32], was observational and did not require invasive techniques. The presence of booted eagle is one of the reasons for which the area is designated as a Special Protection Area under Directive 79/409/EEC on the conservation of wild birds.

### Study Area and Raptor Species

This study of longevity of nesting-platforms was conducted in a mountainous area with elevations ranging from 550 to 1,234 m above sea level, which covers about 10,000 ha of the Special Protection Area “Sierras de Burete, Lavia y Cambrón” (ES0000267), located in the centre of the province of Murcia, southeastern Spain (38°00′ N, 1°45′ W). The climate is dry Mediterranean, with annual precipitation of about 400 mm and a mean annual temperature of 17°C. The mountainous landscape is characterized by a forested ecosystem dominated by one tree species, the Aleppo pine, *Pinus halepensis*, a conifer that reaches up to 22 m in Mediterranean areas [33]. Although the study area contains traditional agroecosystems in the valleys (mostly dry-land crops of vine, olive, almond and cereals), the forest areas, where the breeding territories are located, were not substantially disturbed by human activities (such as timber harvesting) during the study period. The breeding structures studied are nesting-platforms constructed and used by three forest raptors: the booted eagle, the common buzzard and the northern goshawk. The booted eagle is a trans-Saharan migrant, arriving in the study area in late March and leaving in late September [30], while common buzzard and northern goshawk are sedentary in the study area, which represents the southernmost part of their distribution range [34]. Their abundance differs, booted eagle being the dominant species (20–29 breeding pairs), followed by common buzzard (4–12) and northern goshawk (0–4). These breeding pairs defended a total of 70 territories during the study period, defined as any stretch of forest containing one (usually) or several nests (up to seven, within less than 300 m from each other) [20], and forage in undefended hunting grounds up to 17 kilometres distant [35]. The three species have a similar breeding phenology in the study area [23]. Booted eagle females lay one or two eggs, while buzzard females lay one to three eggs and goshawk females lay one to four eggs [34]. The studied species exhibit strong territorial behaviour [24], goshawks being the largest of the three species (booted eagle, body mass ≈ 510–1250 g; common buzzard, body mass ≈ 427–1360 g; northern goshawk, body mass ≈ 517–2200 g; [36]) and the dominant competitor over buzzard in some forests [26], [27]. The goshawk is a rare and endangered species in the study area, but the other two species have a more favourable conservation status with higher population densities [37].

### Nesting-platforms Location and Monitoring

Nesting-platforms were monitored each breeding season, from the end of March to the beginning of May, over a period of 16 years (1998–2013), as part of an intensive long-term monitoring of the occurrence and productivity of the population of the three raptor species studied [22], [29]–[32], [38], [39]. Previous field work in 1996 and 1997 was necessary to locate most nesting-platforms in the study area, so it was assumed that the location of all nests since 1998 was known [22]. In 1998, we found 80 nests, which were systematically monitored until 2013, over which time we also searched for new nests. Nest searching activity is a time-intensive and difficult task in coniferous forest compared to searching cliffs or deciduous forests [40]. In our study area, most nesting platforms remain stable over a number of years and site fidelity of forest raptors [20], [39] facilitated population monitoring. Therefore, the search for nests consisted of locating the territories of the breeding pairs during the courtship period, when species show strong territorial defence (intra- and interspecific), and a subsequent search on foot to find new nesting-platforms. The occupancy of a breeding site was determined when any sign of territorial or mating behaviour was observed, including territorial flights, courtship, responses to mating calls (e.g. elicited vocalizations, approaches), copulations or by direct evidence of breeding (for further details see [22], [29]). All known breeding sites were visited to detect occupancy, as well as other suitable but previously unoccupied areas in the search for newly established territories. Because all three species studied construct nests of similar appearance and dimensions [40], a species must be observed using a brand-new nest to have been considered the builder species. The birds may also reuse old nests, placing new material in the nest, typically pine needles, before laying [20]. For this reason, nest size of an individual nest could vary across years, with an average area ranging from 0.08 to 1.07 m^2^ and a height (from the nest base to the nest cup) ranging from 13 to 88 cm [38]. We had no evidence of nest usurpations between different species in their first year of nest constructions. When a new nest was found, its location was recorded by a GPS unit and incorporated in a geographical information system (GIS). At the same time, information on a set of variables that we considered relevant to the longevity of nesting-platforms [28], [41] and which had been used as microhabitat variables in previous studies was recorded on a circular plot around the nest-tree with a radius of 10 m [22], [32]. These variables and the related hypothesis are presented in Table 1. Variables relating to the characteristics of nesting-platforms (NESTH) and nest-trees (TREEH, DBH and NTCC) were recorded for 123 nests. The nest builder species (SP) was recorded for the 71 nests built during the study period. The frequency of use (FREUSE) was recorded for each nest, as the occupancy of all active nests was known during the study period.

**Table 1:** Definition of variables used in the models to analyse the lifespan of nesting-platforms and establish relationships for nest survival in a Mediterranean forest ecosystem, the Special Protection Area “Sierras de Burete, Lavia y Cambrón”.

Acronym	Definition and unit	Prediction for nest survival a
NESTH	Nest height (m)	+
TREEH	Nest-tree height (m)	+
DBH	Nest-tree diameter at breast height, 1.3 m above the ground (cm)	+
NTCC	Nest-tree crown cover (%)	+
SP	Nest builder species; categorical variable of forest raptors that built the nest (BE: booted eagle/CB:common buzzard/NG: northern goshawk)	CB ≠ NG ≠ BE^b^
FREUSE	Frequency of use of the platform; measured as the proportion of time that the platform was occupied byforest raptor in relation to the number of years that the platform was available, i.e. is considered “live”.	+

a +: an increase in the variable favours nest survival.

b The persistence of the nesting-platform is predicted to be different for each builder species.

A platform was considered to be destroyed when the whole structure had fallen from the nest tree or the branch that maintained it, when the nest tree or nest branches were broken or had fallen, or when most of the nest material (>80%) had deteriorated due to natural causes, mainly meteorological perturbations, resulting in a loss of structure [31].

### Breeding Success Monitoring

For each nest occupied, the breeding success or breeding failure was recorded by the usual methods used for forest raptor census [42]. We made at least three visits to the nests by climbing the nest tree and made observations at a distance by binoculars (x 10) or telescope (x 20–40), see more details in Martínez et al. [22] and Jiménez-Franco et al. [20]. The breeding success was determined by the existence of one or more fledglings in the nest, considering those which survived to about 45 days old [43]. In total, 354 successful breeding attempts were recorded in the studied species for 496 occupancy events.

### Statistical Analysis

#### Rates of nest creation and destruction

In order to obtain the mean annual rate of nest construction, we calculated the number of new nests built by breeding pairs divided by the total number of pairs observed the same year. This rate was estimated for the whole raptor community and for each species separately. A generalized linear mixed model (GLMM) [44] was used to analyse whether the probability of nest building was related to the studied species (booted eagle/common buzzard/northern goshawk). GLMMs are an extension of generalized linear models, which accommodate the dependence between observations within groups (years), considering both random and fixed effects. We considered year as a random effect and the species as fixed effects, using a logit link function and a binomial distribution error. Analyses were performed using the *glmmML* function [45] in the statistical software R, version 3.0.2 [46]. The level of significance for statistical analyses was set at *α = *0.05. The mean rate of nest destruction was calculated from the average rates of nest destruction yearly, that is, the number of nests destroyed each year divided by the total number of nests monitored the previous year. Because the estimation of nest construction and destruction rates of a given year rely on complete census information of the previous year, these rates could not be calculated for the first year of the study (1998).

#### Lifespan analyses

*Kaplan-Meier survival estimates and Cox proportional-hazards regression models:* Kaplan-Meier survival estimates were used to quantify median survival rates of nesting-platforms. Median lifespan was calculated as the age (in years) when survival reached 0.50 [47]. This method allows the inclusion of right-censored data [2], [48], i.e., the inclusion of nesting-platforms, whose exact time of loss is unknown since they persisted until the end of the study. Therefore, the persistence of a nesting-platform (platform lifespan, in years) was calculated from the year the nest was found until the year it was destroyed or until the last census (2013), including those nests constructed before 1998. This pooled analysis prevented us from underestimating the average lifespan, as many of these nests of unknown age (42 out of 86) were still “live” at the end of the study. Cox’s [49] proportional-hazards regression models were used to estimate the effects of nesting-platform characteristics on odds of loss (probability that the nest will not persist to the next year), which is related to longevity. This statistical technique allows the inclusion of covariates and right-censored data. Simple survival models, which included the effect of single covariates, were fitted to test the odds of nesting-platform loss based on the explanatory variables presented in Table 1. Given that the sample sizes varied among explanatory variables, instead of ranking the models in order of parsimony (using Akaike’s Information Criterion, AIC, [50]), we determined whether the models used were statistically significant, setting *α = *0.05. Survival analyses were carried out using the functions *survfit* and *coxph* from the ‘‘survival’’ package [51] of the statistical software R.

**Figure 1:**
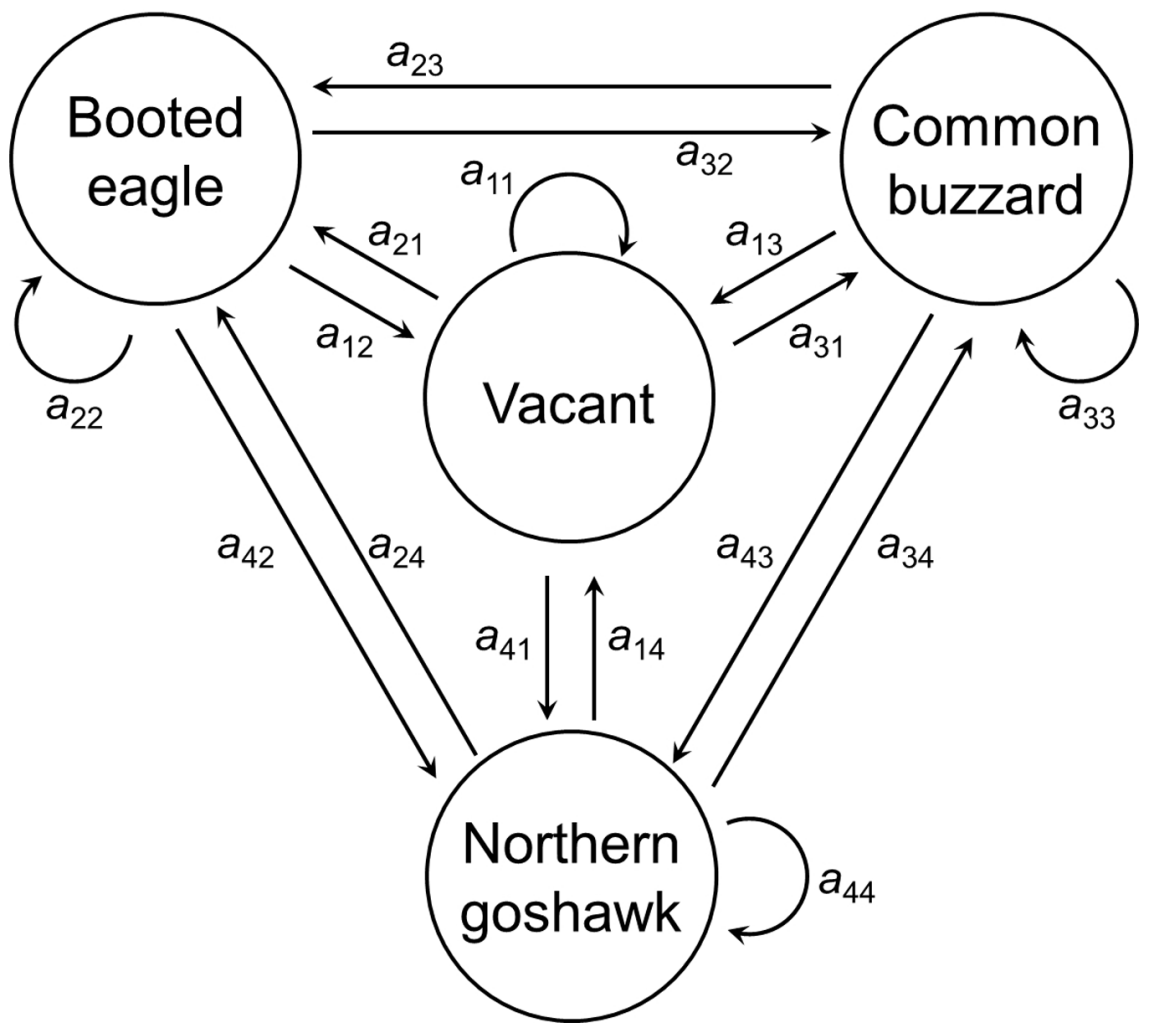
Graph of occupancy stages during the life of nesting-platforms in a Mediterranean forested area. Circles represent different occupancy stages: occupied by booted eagle; occupied by common buzzard; occupied by northern goshawk; vacant nest. Solid arrows represent a possible transition in stage from one year *t* to the next year *t*+1. These transitions are also defined by the element *a_ij_* of the transition matrix **T** (equation 1). Note that nest building is not represented by any nest stage, as they are all built by forest raptors.

*Life expectancy analysis:* We developed an occupancy stage transition matrix, similar to those used in demographic studies with individuals [11], [52], [53] to obtain the life expectancy of a set of nests. Nests were considered as individuals of these demographic studies with a set of occupancy stages during their life (Fig. 1). We defined four life cycle stages as the different occupancy states of each nesting-platform; from 1 to 4, these are: vacant, occupied by booted eagle, occupied by common buzzard and occupied by northern goshawk, respectively. The projection interval was one year since platforms may change their occupancy state in the breeding period every year. Given the projection interval and the stages, a 4×4 time-invariant transition matrix **T** was made for the whole study period. Therefore, the transition matrix **T** represents transitions of the four occupancy states of nests already present in the population after one time step, in which *a_ij_* gives the transition probabilities of extant stage *i* nest at year *t*+1 by a stage *j* nest at year *t*:
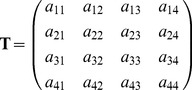
(1)


As **T** is the transient portion of an absorbing Markov chain, with nest destruction as an absorbing state, the column sums of **T** are less than or equal to 1.

Following Caswell & Fujiwara [13], the (*i*, *j*) entry of the fundamental matrix.
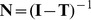
(2)gives the expected number of time intervals spent in stage *j* before nest destruction by a nest starting in stage *i*, where **I** is the identity matrix. The sum of the time spent in all the transient states before final absorption (i.e., the column sums of **N**) gives the vector of life expectancies or average longevity of nests [11]. Given a transition matrix **T** as input, estimates of the fundamental matrix, **N,** mean life expectancies and their variance were obtained using the function *fundamental.matrix* from the ‘‘popbio’’ package [54] of the R statistical software. Standard deviations of the life expectancies are the square roots of the variances.

##### Effects of nest use on breeding success

We used GLMMs [44] to test whether the frequency of use of the nests (variable FREUSE described in Table 1 and determined for each nest along the study period) has an effect on the breeding success of each species. For these analyses we considered each nest as the random variable and the frequency of use as the explanatory, fixed factor. Since probability of breeding success was modelled as a binary variable (1 = breeding success, 0 = breeding failure), a logit link function and a binomial distribution error were used for these models.

## Results

### Patterns of Nest Creation, Distribution and Destruction

**Figure 2:**
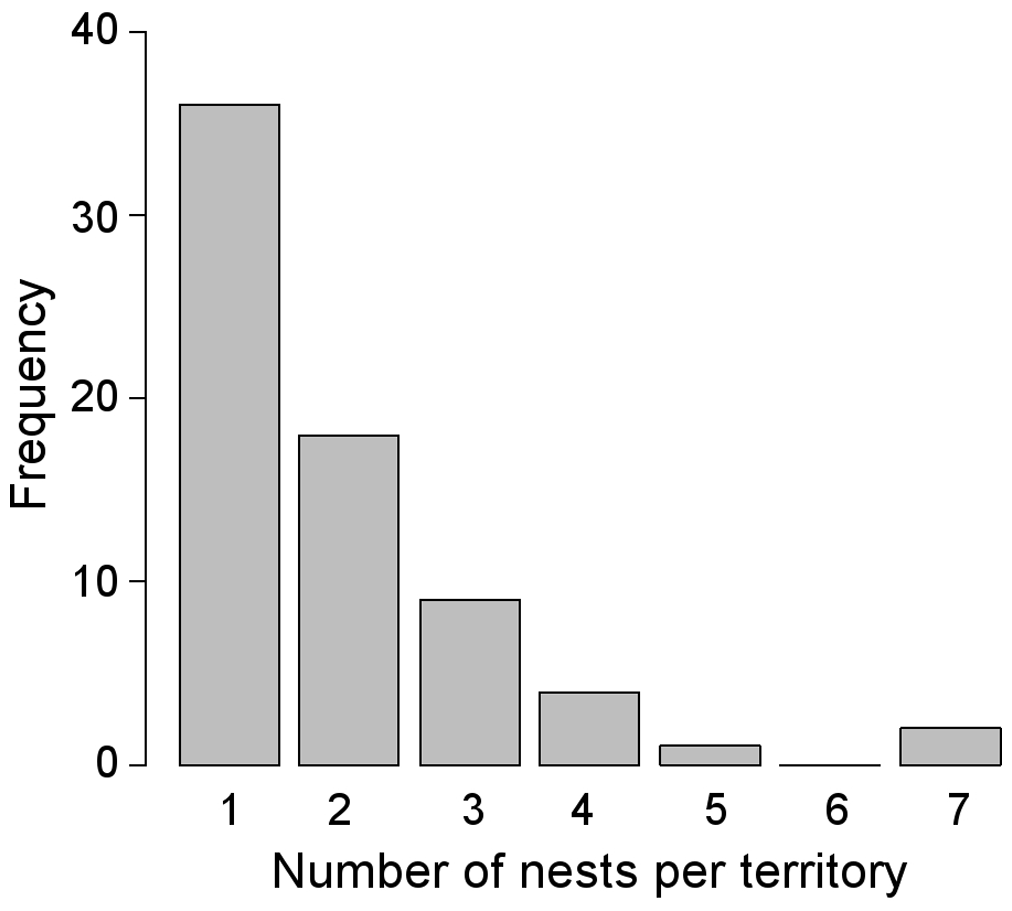
Number of nests per territory in the study area. Distribution of nests (frequency) in the 70 territories in the Special Protection Area “Sierras de Burete, Lavia y Cambrón”.

From a total of 1330 observations in 157 nesting-platforms monitored during the study period, we recorded a low rate of nest construction (Table 2), with an average of 0.14 (±0.09) new nests per year from 1999. The mean rates of nest building by species show that northern goshawk had a higher rate of nest building than booted eagle and common buzzard (0.35±0.09, 0.13±0.09 and 0.10±0.09, respectively). There were significant differences in the nest building rates between booted eagle and northern goshawk (*P*<0.001), and between common buzzard and northern goshawk (*P*<0.001), but not between booted eagle and common buzzard (*P = *0.469). The 157 nesting-platforms were distributed among a total of 70 territories in the study area: 51.4% territories were constituted by one nesting-platform, 25.7% territories by two nests, and the rest of the territories (22.9%) by three or more nests (Fig. 2). Although 56.1% nests were destroyed between 1999 and 2013, 88 nests were present at the end of the observation period (Table 2). The average rate of nest destruction calculated from 1999 onwards was 0.05 (±0.04) nests per year, and was highest in 2007 and 2008 (Table 2) as a result of a severe storm in January 2007 [31].

**Table 2:** Number of nests from 1998 to 2013 in the study area “Sierras de Burete, Lavia y Cambrón”, considering their different occupancy states by raptors (booted eagle, common buzzard, northern goshawk), nest construction events and nest destruction events.

	1998	1999	2000	2001	2002	2003	2004	2005	2006	2007	2008	2009	2010	2011	2012	2013
**Total of nests**	80	82	83	84	84	84	90	91	96	93	86	91	94	90	93	88
Vacant	43	48	54	53	46	54	56	60	64	58	49	61	60	57	61	55
Occupied by booted eagle	29	26	21	23	26	22	25	21	21	22	21	20	23	23	24	25
Occupied by common buzzard	6	7	6	8	9	6	8	7	8	9	12	7	8	7	4	6
Occupied by northern goshawk	2	1	2	0	3	2	1	3	3	4	4	3	3	3	4	2
**Total of built nests**	_	2	1	1	5	3	6	5	5	7	4	7	6	4	12	3
Built by booted eagle	_	2	0	1	3	2	5	3	2	4	1	4	3	3	9	2
Built by common buzzard	_	0	0	0	0	1	1	1	2	1	2	1	1	0	1	0
Built by northern goshawk	_	0	1	0	2	0	0	1	1	2	1	2	2	1	2	1
**Total of destroyed nests**	_	0	2	0	5	3	1	4	1	10	13	1	4	8	9	8

### Nest Persistence

The median lifespan (when survival reaches 0.50) for all monitored nesting-platforms built by the three studied species in Aleppo pines was 12 years (Table 3). None of the models fitted to predict hazard of nest loss in relation to the characteristics of the nest and nest-tree, nest builder species and frequency of use of the platforms, was significant (*P*>0.05; Table 4).

**Table 3:** Survival of nesting-platforms in a Mediterranean ecosystem in the Special Protection Area “Sierras de Burete, Lavia y Cambrón” (southeastern Spain) based on Kaplan-Meier estimates.

Nest age (yr)	No. nests at risk^a^	No. Censored^b^	No. event (No. lost)^c^	Survival rate	S.E.	lower 95% CI	upper 95% CI
1	157	3	1	0.994	0.006	0.981	1.000
2	153	12	4	0.968	0.014	0.940	0.996
3	137	3	1	0.961	0.016	0.930	0.992
4	133	6	10	0.888	0.026	0.838	0.942
5	117	5	4	0.858	0.030	0.802	0.918
6	108	3	6	0.810	0.034	0.747	0.879
7	99	5	9	0.737	0.039	0.665	0.816
8	85	6	7	0.676	0.042	0.599	0.763
9	72	1	7	0.610	0.044	0.529	0.704
10	64	3	9	0.524	0.046	0.441	0.624
12	52	0	2	0.504	0.047	0.420	0.605
13	50	0	2	0.484	0.047	0.400	0.586
14	48	1	4	0.444	0.047	0.360	0.547
15	43	40	3	0.423	0.047	0.340	0.527

a Number of usable nests monitored up to a given age.

b Censored nests were still standing at the end of the study.

c Number of nests that were lost (through natural causes).

**Table 4:** Model selection results based on Cox proportional-hazard models of hazard of loss in relation to nest characteristics (model 1), nest tree characteristics (models 2–4) and nest-occupancy variables (model 5–6) for the 157 nesting-platforms in the Special Protection Area “Sierras de Burete, Lavia y Cambrón”, southeastern Spain.

Model^a^	Coefficient	Hazard ratio (*e* ^coef^)^b^	SE of the coefficient	*z*	*P*
1. NESTH	*β*_1_ = −0.13	0.88	0.07	−1.89	0.06
2. TREEH	*β*_1_ = −0.03	0.97	0.05	−0.58	0.56
3. DBH	*β*_1_ = −0.005	1.00	0.00	−1.09	0.28
4. NTCC	*β*_1_ = 0.002	1.00	0.01	0.21	0.84
5. SP^c^	*β*_buzzard_ = −0.79	0.46	0.62	−1.27	0.21
	*β*_goshawk_ = −0.08	0.92	0.51	−0.16	0.87
6. FREUSE	*β*_1_ = 0.07	1.08	0.40	0.19	0.85

a NESTH: nest height; TREEH: height of the nest-tree; DBH: nest tree diameter; NTCC: crown cover of the nest tree; SP: type of nest building forest raptor species; FREUSE: frequency of use of the nest. Variables are described in Table 1.

b The hazard ratio is equal to exp (estimated coefficient) and represents the change in hazard per unit compared to a baseline hazard rate. A hazard ratio of 1 indicates no change in hazard, a hazard ratio above 1 indicates an increase in hazard (shorter lifespan), and below 1 indicates a decrease (longer lifespan).

c The builder species booted eagle was considered as the “control” with which the other two builder species were compared.

The expected longevity of nests or life expectancy obtained from the transition matrix **T** ranged from 17.94 to 19.72 years, depending on the different occupancy states (Table 5). Standard deviations of longevity were high but varied little between the different occupancy stages. The complete transition matrix obtained from a time-invariant model for all the nest stages, **T**, shows a high probability of transition from the vacant stage in year *t* to the same stage in year *t*+1 (Table 5). Probabilities of nest reuse by raptors (diagonal values) were higher in booted eagle than in common buzzard and northern goshawk, respectively (Table 5). The first column of the fundamental matrix, **N**, corresponds to vacant nests, and, on average, a vacant nest will be 13.19 years in a vacant stage, 3.32 years in a stage occupied by booted eagle, 1.16 years occupied by common buzzard and 0.27 years occupied by northern goshawk (Table 5).

**Table 5:** Transition matrix **T** among different occupancy states, fundamental matrix **N** and life expectancy of nesting-platforms in each occupancy state (vacant, booted eagle, common buzzard and northern goshawk) for the study period (1998–2013).

Transition matrix (T, 1998–2013)				
year *t*+1	year *t*			
	Vacant	Booted eagle	Common buzzard	Northern goshawk
Vacant	0.808	0.289	0.342	0.658
Booted eagle	0.080	0.636	0.117	0.079
Common buzzard	0.034	0.046	0.477	0.026
Northern goshawk	0.015	0.009	0.000	0.184
**Fundamental matrix (N)**				
	**year ** ***t***			
**year ** ***t*** **+1**	**Vacant**	**Booted eagle**	**Common buzzard**	**Northern goshawk**
Vacant	13.189	12.203	11.376	12.185
Booted eagle	3.323	5.908	3.501	3.364
Common buzzard	1.162	1.328	2.973	1.162
Northern goshawk	0.270	0.280	0.240	1.478
**Life expectancy**	17.944	19.719	18.090	18.189
Standard deviation	17.8	17.9	17.8	17.8

Values of fundamental matrix and life expectancy are expressed in years.

### Patterns of Nest Use and Alternation

**Figure 3:**
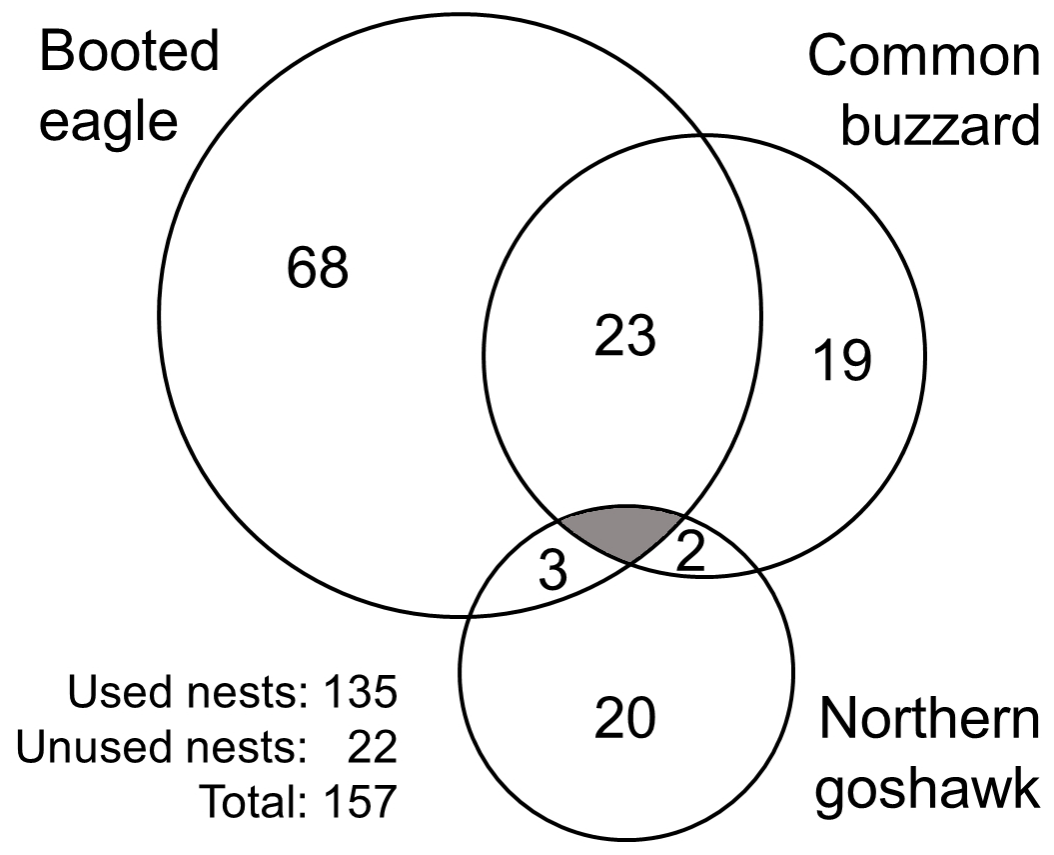
Nest use pattern by the three species (booted eagle, common buzzard and northern goshawk). Circles represent number of nesting-platforms occupied in at least one breeding attempt by each species during the study period (1998–2013). Note that some nests were alternately occupied by two species but no nest was shared by the three species (shaded area).

Most of the nesting-platforms (135, 86.0%) were occupied in at least one breeding attempt during the study period (Fig. 3). Of the total number of nests monitored, 106 (67.5%) were reused. The 22 unused platforms were nests built before 1998. Most platforms were occupied for at least one breeding attempt by booted eagle (59.9%), the most abundant species in the study area (Table 2), followed by common buzzard (28.0%) and northern goshawk (15.9%). Moreover, 17.8% of all nests were used alternately in different breeding periods during the study period (14.7% between booted eagle and common buzzard, 1.9% between booted eagle and northern goshawk and 1.3% between common buzzard and northern goshawk), although no nest was shared by all three studied species (Fig. 3).

**Figure 4:**
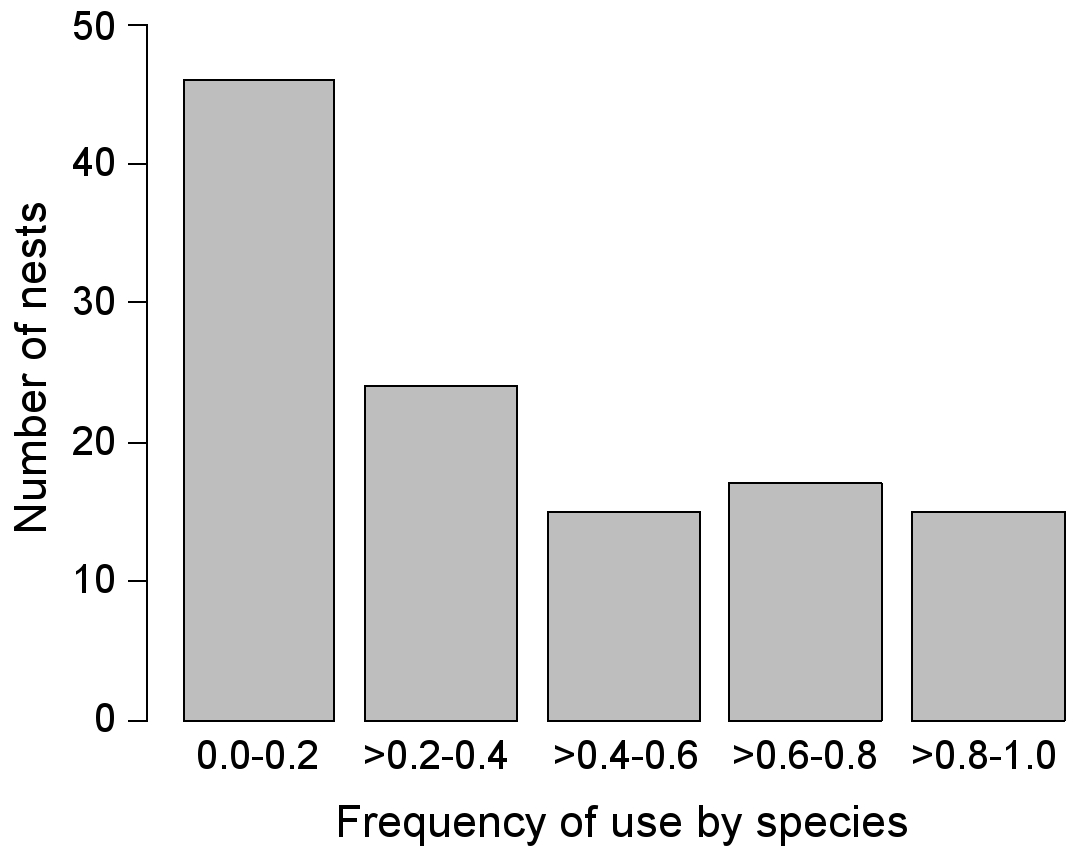
Frequency of use of nesting-platforms by forest raptors during the study period (1998–2013). Only nests that were available for 5 or more years were considered (*n* = 117).

Nests were occupied in a mean of 3.31 breeding attempts (±3.22; *n = *157). The average frequency of nest use (i.e., the number of occupancies in relation to the number of years that the nests were available), considering only nests that were available for five or more years, was 0.39 (±0.31, *n = *117); more than a half of the nests had a low frequency of occupancy (Fig. 4).

### Effects of Nest Use on Breeding Success

The frequency of nest use did not have a significant influence on the probability of breeding success of any of the studied species (booted eagle: *β = *0.81, *n = *346, *P = *0.316; common buzzard: *β = *0.30, *n = *112, *P = *0.736; northern goshawk: *β* = −0.97, *n = *38, *P = *0.399).

## Discussion

The mean rates of nest construction and loss were low. Forest-dwelling raptors tend to create nests in large, often old trees, and it is difficult to increase the availability of such nests [28], unlike in the case of other bird populations with a shortage of nests, where the installation of nest boxes is a successful management tool for conservation [7], [8]. Furthermore, artificial nest-site provisioning in the case of forest-dwelling raptors may not improve their breeding success, as these nests may function as ecological traps [55]. Although a shortage of nest sites is not a known problem for the raptors in this community, these nesting-platforms are the only breeding stands for the occupant raptors in the study area and nest construction rates are low. Therefore, we suggest that these breeding structures are important resources for the conservation of forest raptors. Unlike for cliff-nesting raptors such as golden eagle *Aquila chrysaetos*, where most territories have 5 or more nests [5], or bearded vultures *Gypaetus barbatus* that can have more than 10 nests [4], our results show that most territories were constituted by only one nesting-platform. In other species, such as the Spanish imperial eagle *Aquila adalberti*, the average number of nests per territory was 3.5, a figure that increased with the number of years the territory was occupied, from 2.5 nests per territory in territories monitored for 5 years to 5.6 nests per territory for those monitored for more than 16 years [56]. These findings highlight the importance of conserving these nests in order to maintain a large number of breeding sites active for the forest raptor community. In this context, where the conservation of nesting-platforms is a key action for the viability of populations, especially when species depend on these limited resources [55] and they may be affected by meteorological perturbations [31], [57], we suggest that estimating the longevity of breeding sites is a useful tool for the conservation and management of these species.

In order to calculate the permanence of nests, we not only applied the Kaplan-Meier survival estimates used in previous studies [2], but also, for the first time in the literature, estimated the life expectancy for each occupancy state of the nests by using a transition matrix [11]. These estimates were made using stage-classified models, considering nesting-platforms to be similar to an individual that undergoes several stages during life (in the present case, the stage is the nest occupancy state). In our study, the estimates of nesting-platform survival obtained with both methods showed complementary aspects. While the Kaplan-Meier survival estimate provided the median longevity of nests (around 12 years), the population matrix model provided the average life expectancy for each of the possible states of nest occupation; for this reason, survival estimates were higher with the second kind of lifespan analysis (ranging from 17.94 to 19.72 years for vacant and occupied by booted eagle, respectively). Standard deviations of life expectancy were high and similar to the survival estimates (e.g. 17.94±17.8 years in the vacant stage), indicating that some nests show higher permanence values than others. Our results show that the survival estimates for the nesting-platforms, in general, were high and in accordance with the occupancy patterns of the breeding populations, as nests in the study area were occupied for a mean of 3.31 breeding attempts. These results for nest longevity agree with previous studies in the raptor community: high fidelity to the breeding sites, consecutive occupancies by booted eagle in the same territory ranging from 1 to 6 years [39], and reoccupations by pairs of booted eagle and common buzzard, with around 85% reuse of the same nests [20]. We suggest that, although a nest may remain empty for several years, its structure should be retained for its importance in raptor reproduction. Although there is no information concerning the longevity of nesting-platforms built by forest raptors in other areas, our median estimate is similar to estimates obtained for other kinds of breeding sites, such as tree holes [2], [14] and temporary ponds [58]. However, nest persistence in the present study was higher than that of nests built by European magpies and used by forest raptors, which persist for around 3 years [1] and lower than in nests created on cliffs, which may remain for hundreds of years [15].

The Cox proportional-hazards regression models used to test whether the persistence of nesting-platforms is related to nesting-platform height, nest-tree height, nest-tree diameter, crown cover of the nest-tree, the nest builder species and the frequency of platform use were not significant. These results suggest that the habitat and nest-trees are sufficiently homogeneous for nests to be persistent, as the Aleppo pine is the only tree species that supports the nesting-platforms of raptors and has a good protective crown cover [28]. Moreover, these results agree with those of Pagán et al. [32], who indicated that territorial occupancy of the study area does not follow any occupancy pattern related to nest-trees and other habitat characteristics. However, another study showed that the persistence in tree cavities in a Canadian forest increased with the nest-tree diameter [2].

In our study, the nesting-platforms are placed in only one tree species, the Aleppo pine, but future studies should be directed at estimating the longevity of raptor nesting-platforms in different types of forest tree species. For instance, the persistence of cavities located in heterogeneous forest systems may also be influenced by the type of tree species [14] or forest habitat [2]. The next step could consist of determining the relationship between the longevity of nesting-platforms and tree longevity. Along these lines, other studies developed in cavities showed that cavity longevity is related to the stage of decay of the nest tree [2], [59]. The second kind of lifespan analysis used in this study, which involved a transition matrix, could be extended to other management actions. Just as in population demography it is useful to simulate the viability of populations [52], [60], a potential future step may be to simulate the effects of creating artificial nests (after incorporating the rate of artificial nest creation as a parameter of fertility in the model) [11] in order to ascertain the temporal dynamic of the nests and its effect on the abundance of the forest raptor community.

The results of describing the patterns of nest reuse and alternation among forest raptors show that most nesting-platforms (86%) were occupied for at least one breeding attempt during the study period and some nests were alternately occupied by different breeding species. These results support previous studies which indicate that nests are frequently reused by forest raptors since they may act as location cues in the process of territorial settlement [20]. Nesting-platforms in the present study showed similar average reuse rates to magpie nests reused by a forest raptor community [1] (3.31 *vs*. 1.13 times, respectively). The frequency with which nests were reused for 5 or more years varied, suggesting that some nests were vacant most of the breeding attempts and other nests were frequently reused. The reuse of nest-sites has important consequences for the viability of populations. As nest building is an energetically costly task, reusing an old nest may improve breeding success [61]. On the other hand, old nests are more likely to carry diseases or ectoparasites [5], [62]. However, our results show that the frequency of nest use did not have a significant effect on the breeding success of any of the studied species. These results are in agreement with those of a previous study, in which the reproductive success of pairs of booted eagle and common buzzard did not differ significantly between pairs reusing an old nest and pairs that built a new one [20].

In summary, the fact that the study area is not affected by human perturbation provides a good opportunity to analyse the patterns of nest creation and destruction, nest persistence and reuse by forest raptors in a Mediterranean forest ecosystem. We conclude that nesting-platforms are important breeding resources for the occupancy and viability of the studied forest raptor community and should be conserved since nest construction rates are low, most of the nests constitute a territory with only one nest and nests persist for a sufficiently long time to permit the forest raptor community to enjoy a high rate of nest reuse and alternation. In short, all the forest nesting-platforms should be kept in order to preserve an adequate supply of breeding sites for the raptors in our study area.
